# Acceptability of Audiovestibular Assessment in the Home—A Patient Survey

**DOI:** 10.3390/audiolres14030045

**Published:** 2024-06-20

**Authors:** Amanda J. Male, Nehzat Koohi, Sarah L. Holmes, Robert D. S. Pitceathly, Diego Kaski

**Affiliations:** 1 SENSE Research Unit, Department of Clinical and Movement Neurosciences, Institute of Neurology, University College London, 33 Queen Square, London WC1N 3BG, UK; 2 NHS Highly Specialised Service for Rare Mitochondrial Disorders, Queen Square Centre for Neuromuscular Diseases, The National Hospital for Neurology and Neurosurgery, London WC1N 3BG, UK; 3 Department of Neuromuscular Diseases, UCL Queen Square Institute of Neurology, London WC1N 3BG, UK

**Keywords:** COVID-19, home visit, audiological assessment, survey

## Abstract

The COVID-19 pandemic dramatically changed health service delivery with vulnerable patients advised to isolate and appointments provided virtually. This change affected recruitment into an observational cohort study, undertaken at a single site, where participants with mitochondrial disorders were due to have specialist hospital-based audiovestibular tests. To ensure study viability, the study protocol was amended to allow home-based assessment for vulnerable participants. Here, we report outcomes of an online survey of participants who underwent home-based assessment, related to the experience, perceived benefits, and drawbacks of home audiovestibular assessments. Seventeen participants underwent home-based neuro-otological assessment, due to the need to isolate during COVID-19. Following the assessment, 16 out of 17 participants completed an anonymised online survey to share their experiences of the specialist home-based assessment. One hundred percent of participants rated the home-based assessment ‘very positively’ and would recommend it to others. Sixty-three percent rated it better than attending hospital outpatient testing settings. The benefits included no travel burden (27%) and reduced stress (13%). A majority reported no drawbacks in having the home visit. The patient-reported feedback suggests a person-centred approach where audiovestibular assessments are conducted in their homes is feasible for patients, acceptable and seen as beneficial to a vulnerable group of patients.

## 1. Introduction

Mitochondrial diseases are a diverse group of genetic disorders caused by mutations in nuclear-encoded and mitochondrial DNA (mtDNA)-encoded genes [1]. Adults with primary mitochondrial disease (PMD) present with a diverse range of symptoms and features, including diabetes, neuropathy, retinitis pigmentosa, hearing and vestibular impairment, ataxia, myopathy, cardiomyopathy, and/or fatigue.

The implications of these symptoms are profound. They can affect patients’ quality of life, undermine participation and independence in daily activities, and impact cognitive functions. Fatigue can also be exacerbated—a common and significant challenge reported by up to 100% of individuals with PMD [2]. Dizziness and unsteadiness are common in PMD, as a result of neuropathy and ataxia, but also a combination of peripheral and central vestibular dysfunction [3]. The identification of vestibular impairment in PMD offers a unique treatment opportunity given the extensive evidence supporting vestibular rehabilitation for both symptom reduction and to improve functional outcomes [4,5,6,7,8]. Waiting lists for specialist neuro-otology testing can contribute to delays in gaining a diagnosis, and in turn accessing the appropriate treatment for dizziness and unsteadiness.

A decision-making framework was developed in collaboration with patient and public involvement (PPI) and following a modified Delphi approach [9] to facilitate the identification of vestibular pathology in patients with PMD. This decision-making framework was then tested for sensitivity and specificity in a tertiary outpatient vestibular neurology clinic and then in an observational study [10]. Ethical approval was obtained in February 2020. As part of this observational study, participants would have a battery of audiovestibular assessments completed when they attended the tertiary hospital for routine medical or therapy appointments.

The COVID-19 pandemic was a global health crisis caused by severe acute respiratory syndrome coronavirus 2 (SARS-CoV-2). It resulted in severe social and economic disruption around the world and suspended all outpatient hospital appointments and research activity. Adults with PMD were identified as being a vulnerable group [11] and some adults were subsequently advised to continue to isolate once research was able to resume.

To ensure this study remained viable, the research team decided to amend the study design, following appropriate ethical approval, such that audiovestibular assessments could be completed in the participants’ homes instead of in the hospital, when deemed necessary. Home visits included researcher testing for possible COVID-19 and followed infection control procedures to minimise the chances of cross-infection. The provision of home-based assessments was anticipated to mitigate participant fatigue by obviating the need for travel to appointments, potentially enhancing the patient experience.

Providing specialist care in the home instead of a hospital setting has been researched and implemented following cardiac surgery [12], in post-natal care [13], after psychosis [14], with cardiac rehabilitation [15], and for severe pneumonia in children [16]. Home-visiting services are ideally suited to individuals who are unable to visit the clinic due to medical or logistical reasons. Audiological home visits are offered by some independent audiology or hearing care providers (e.g., Hearing Aid UK and Specsavers) who travel to the patient’s home to conduct assessments, hearing aid fittings, or adjustments for hearing aids, and provide necessary care or even counselling. Such services have not been validated against in-store assessments and presumably constitute basic audiometric testing for hearing aid provision or adjustment. Furthermore, to our knowledge, no studies have explored the feasibility of providing a specialist (tertiary level) assessment in the home instead of the hospital, nor of providing neuro-otology assessments specifically in the home. This suggests a novel opportunity for specialist services to explore alternative ways of delivering care, especially to vulnerable groups, who may otherwise forsake such assessments that may help identify targeted treatment options.

Herein, we assessed the feasibility and patient experience of receiving specialist audiovestibular assessments at home during the COVID-19 pandemic. Through this investigation, we hope to contribute to the broader discussion on patient-centred healthcare delivery in the context of evolving public health challenges.

This paper aims to understand the participants’ experiences of receiving this specialist assessment in the home, as part of a larger observational cohort study.

## 2. Materials and Methods

### 2.1. Recruitment and Participants

Adult participants (16 years of age or over) attending the specialist mitochondrial disease clinic in London, with a genetically confirmed diagnosis or clinicopathological diagnosis of PMD, were recruited as part of a larger observational study. The full details of this study are published elsewhere [10]. Participants could only participate if they were negative for COVID-19 screening, and therefore needed to complete a COVID-19 screen and temperature check, as per NHS guidelines. In relation to the association between the SARS-CoV-2 virus and audiological dysfunction [17,18], we acknowledged the importance of excluding COVID-19 infection in our patients. We could not, however, exclude the presence of asymptomatic infection. The recruitment phase spanned from December 2020 to March 2022. Ethical approvals were obtained (Health Research Authority and Health and Care Research Wales Approval 20/YH/0014).

### 2.2. Home-Based Audiovestibular Assessment

In response to the COVID-19 pandemic, seventeen participants classified as medically vulnerable and unable to attend hospital appointments had assessments conducted in their homes. These assessment sessions lasted around two hours and were facilitated by an experienced research audiologist (NK) with over 18 years of experience. All test equipment was calibrated and maintained, and the tests were conducted in line with routine clinical care protocols. Although the testing time was comparable to clinic-based assessments, additional time demands included travel, which ranged from 20 min to 4 h, and equipment management, requiring an extra 30 min per visit. Each assessment comprised a detailed battery of audiovestibular tests, as outlined in Table 1. This was the same protocol as the test conducted in the hospital setting.

### 2.3. Participant Experience Survey

The seventeen participants assessed at home were subsequently invited to complete an anonymised electronic survey of this experience. The survey was sent out by another member of the research team, separate to the research audiologist, ensuring the responses remained confidential. The lead author, who was not involved in the data collection process, completed the analysis of the responses to control for any potential courtesy bias. The survey was developed in line with clinical service evaluation forms to explore participants views, thoughts, and experiences of having assessments at home. The survey comprised six questions. Three questions collected categorical data, with two questions using a 5-point Likert scale, and three questions collected open-text responses, as detailed in Table 2.

### 2.4. Data Management and Analysis

The data were collected and stored in REDCap (Research Electronic Data Capture), which is an online, secure data collection tool. The data were then exported to Excel 365 for analysis. Responses to the single-choice questions were analysed descriptively and presented as frequencies and percentages. Data from open-text answers were analysed by manifest content analysis [19]. Individuals could give more than one answer, composed of short sentences or single words. Similar words and short sentences were then grouped and presented as frequencies. These groups of responses were then collapsed further into categories for the discussion, when single words or short sentences had related responses.

## 3. Results

### 3.1. Survey Completion and Demographics

Ninety-four percent of the participants (*n* = 16/17) completed the online survey. The age range was 37 to 81 years (mean 58 years; ±SD 12.67 years), with a female-to-male ratio of 9:7. Sixty-three percent of participants that had a home assessment had a diagnosis of m.3243A>G, *MT-TL1* (*n* = 10/16). The remaining six participants had a nuclear mutation (37%), of which two had a clinicopathological diagnosis of mitochondrial disease. Please see Supplementary Information Table S1 for more demographic details.

### 3.2. Home Visit Satisfaction

Of the 16 participants who completed the survey, 100% rated the home visit as ‘very positive’ and would recommend this type of assessment to other patients. When asked how the home assessment compared to their other experiences of hospital assessments, 63% (*n* = 10/16) of participants reported it was ‘better’ and 37% (*n* = 6/16) stated that it was ‘as good’.

### 3.3. Perceived Benefits of Home-Based Assessment

**Figure 1:**
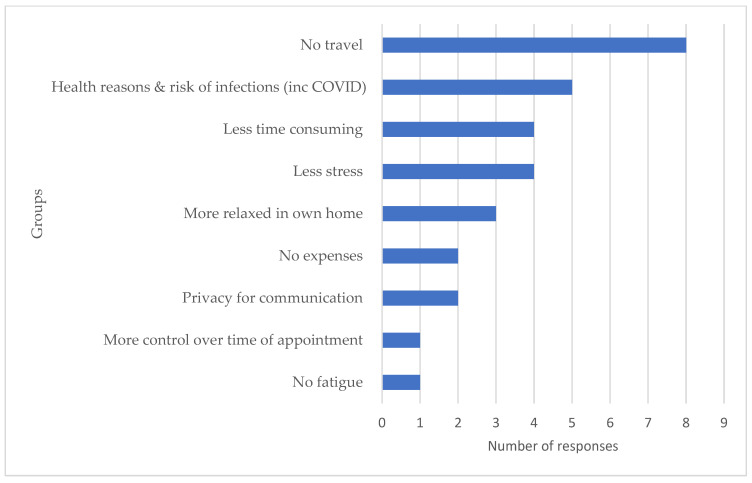
Groups and number of responses for the reported benefits of a home visit assessment.

Participants were able to offer multiple open-text answers on the perceived benefits of a home-based assessment. Figure 1 shows the 9 groups of responses that emerged from the 30 collective responses.

### 3.4. Drawbacks and Limitations

A majority (69%, *n* = 11) reported no drawbacks in having the home visit. However, a small fraction expressed concerns regarding the audiologist’s travel and equipment setup (12%, *n* = 2), and some indicated that not all assessments could be completed at home (19%, *n* = 3).

### 3.5. Test Repetition and Consistency

Of the 16 surveyed participants, 44% (*n* = 7) underwent repeated tests in the hospital as a part of their routine clinical care. The age range was 43 to 73 years (mean 58 years; SD = 11.46 years), with a female-to-male ratio of 4:3. All of these participants had consistent outcomes across both settings. Table 3 outlines the test results for participants who underwent both home and hospital testing.

## 4. Discussion

This survey indicated overall very positive feedback, supporting the feasibility and acceptance of home-based audiovestibular assessments for vulnerable adults with PMD. Participants perceived the home visit as ‘as good’ if not ‘better’ than previous experiences of attending a hospital for assessment and would recommend it to others. The majority also reported no significant drawbacks.

There are benefits to the healthcare service by providing care at the home, specifically reducing the demand on valuable and often limited clinical space. Of relevance, when face-to-face clinic services did resume after COVID-19, there were additional social-distancing measures in place for some time. This reduced the volume of in-person consultations available and resulted in most care being provided either by telephone or as video consultations. For PMD patients, often affected by hearing impairments, the limitations of remote consultation methods are particularly relevant, reinforcing the need for in-person assessments.

Furthermore, it is not possible to complete diagnostic audiovestibular assessments using a virtual method. Thus, if a person could not attend the hospital, then they would not have access to assessment or an explanation for their dizziness or unsteadiness, nor obtain referrals for vestibular rehabilitation treatment. Access to care remains a challenge across medical disciplines, differentially and negatively impacting individuals who are more vulnerable. Along similar lines, health equity is achieved by removing any systematic differences in the use and outcomes among groups regardless of whether these differences result from financial or other barriers to care.

Providing some investigative intervention or contact in the persons home could mitigate the long waiting times that transpire in face-to-face clinic sessions and ensure a regular review of all individuals with progressive conditions is maintained.

The responses reported as benefits of a home assessment were grouped into three categories for discussion, relating to (i) logistics, (ii) feelings, and (iii) burden.

### 4.1. Logistics

Minimising the need for travel and reducing the health risks of coming into contact with others in the general public had the highest number of responses as the benefits of a home visit. This is unsurprising as this survey was completed at a time when little was known about the COVID-19 virus, and the health implications for vulnerable individuals advised to isolate. Prior to each visit, the research audiologist followed a formal risk assessment protocol (UCL), completed the COVID-19 screening, donned the necessary personal protective equipment (PPE), and thoroughly sanitised all equipment. Participants were informed of these meticulous safety measures and reported no health concerns related to having a single clinician visit their home.

### 4.2. Feelings

Participants reported less stress, being more relaxed, having more control over the time of the appointment, enhanced privacy for communication, and feeling more relaxed by having the appointment at home. Some of these issues could have been raised in part due to the COVID-19 situation, but could also reflect concerns over travelling to a tertiary centre with a multi-system neurological condition. As such, they are important factors to consider when designing and delivering person centred care.

### 4.3. Burden

Completing the assessment in the participants home resulted in no expense to the participant and was less time consuming for the participant. One participant also reported no fatigue as a result of the assessment occurring in their home. These factors could compound those mentioned above in the ‘Feelings’ category and result in an increased risk of non-attendance to tertiary care hospital appointments.

Whilst the participant had less expense and time burden, these issues were instead shifted to the service provider, in this instance the research team. Testing equipment needed to be transportable and set up for each assessment, with a vehicle being hired for transportation, and more time required by the research audiologist to complete the tests per participant. Fortunately, the research team could redistribute grant funds to cover the additional expenses, and time was not as pressured as within the health service.

Thirty-one percent of participants reported concerns about the logistics of equipment transportation and setup by the research audiologist, as well as occasional equipment malfunctions.

A thorough home visit risk assessment completed in advance and adherence to a lone working policy ensured the safety of the research audiologist. While minor logistical challenges such as manageable traffic delays, travel distances, and rare equipment malfunctions were noted by the research audiologist, they did not significantly impede the effective conduction of assessments. This experience highlights the importance of being selective in what tests are completed in the home when considering this in future business cases. Furthermore, it may be useful to survey a group of audiologists in order to understand their experiences of conducting home-based assessments instead of clinic-based ones.

Technical difficulties with equipment, particularly during electrophysiological tests such as ABR and cVEMP, were occasionally exacerbated by electrical noise within home environments. However, it is important to note that such issues are not exclusive to home settings and can also arise within hospital environments. Despite these minor issues, the consistency of the test results was confirmed, as participants who underwent subsequent hospital-based testing showed outcomes that mirrored those obtained during home assessments. The main observational study recruited patients with and without dizziness and unsteadiness in order to test the accuracy of the diagnostic framework, and so not all those who had home assessment required routine audiovestibular input to have access to further testing. The reason for including these data, for those that did undergo reassessment, was to reassure readers that the tests that were repeated had similar findings, thus demonstrating that the tests completed at home were reliable and useful.

### 4.4. Limitations

This study did not seek to perform an economic evaluation of home assessment against hospital audiovestibular evaluation. This survey was designed to explore the overall feasibility and acceptability by patients of a home visit method of care. It was undertaken in response to the changing landscape during COVID-19, in order to complete a charity-funded research study. Future studies should consider the costs related to equipment and staff transfer, and insurance costs. The numbers were also small as the study recruitment period came to conclusion. Therefore, a larger study that includes a cost–benefit analysis would need completing to assist in any future business case development for home visit assessment, and to ensure the results are generalisable to the clinical setting.

## 5. Conclusions

The results of this survey suggest that home audiovestibular assessments were feasible and perceived as acceptable and beneficial to a vulnerable group of patients with PMD. Our findings demonstrate that such an approach lessens patient burden. However, the possible shift of logistical and financial responsibilities towards the provider necessitates strategic planning to ensure sustainability. A potential hybrid model, which allows specialists to conduct assessments in patients’ homes or at local community centres/hubs might offer a more tailored and efficient solution, bridging the gap between generalist and specialist care paradigms.

## Figures and Tables

**Table 1:** Details of the audiovestibular tests completed in the home.

**Auditory system**
Pure-Tone Audiogram (PTA)
Tympanometry
Speech in Noise Tests
Auditory-Evoked Brainstem Response (ABR)
Otoacoustic Emissions (OAEs)
**Vestibular system**
Video-Nystagmography (VNG)
Video Head Impulse Test (vHIT)
Cervical Vestibular-Evoked Myogenic Potential (cVEMP)
Dix–Hallpike and Roll Tests (positional tests)
**Visual dependence**
Subjective Visual Vertical (SVV) Test with Virtual Reality

**Table 2:** Participant Experience Survey: Questions and Response Options.

Nos.	Question	Answer Options
1	Overall, how would you rate the home visit assessment?	Very positive = 1 2 3 4 Very negative = 5
2	How did the home visit assessment compare to your expectations?	Better than expected = 1 2 3 4 Worse than expected = 5
3	Would you recommend this type of assessment to other patients?	Yes No Maybe
4	What were the benefits of having a home visit assessment instead of a hospital clinic appointment?	Open-text
5	What were the drawbacks to having a home visit assessment instead of a hospital clinic appointment?	Open-text
6	Finally, do you have any other feedback for us?	Open-text

**Table 3:** Comparison of test outcomes when completed in the home and in the hospital.

Genetic Diagnosis	Time between Tests (Months)	Test Result Comparisons
m.3243A>G, *MT-TL1*	5	PTA (within ±10 dB) LiSN-S (same pattern) cVEMP (same, present) ABR (same, absent)
m.3243A>G, *MT-TL1*	6	PTA (within ±10 dB) cVEMP (same, present)
m.3243A>G, *MT-TL1*	9	PTA (within ±10 dB) cVEMP (same, present)
m.3243A>G, *MT-TL1*	10	PTA (within ±10 dB) ABR (same, absent) VNG (same) DPOAE (same, absent)
m.3243A>G, *MT-TL1*	10	PTA (within ±10 dB) LiSN-S (same pattern) ABR (same, waves I and V were present with normal latencies, whereas wave III and the interpeak latencies I–III were delayed) cVEMP (same, present)
m.3243A>G, *MT-TL1*	11	PTA (within ±10 dB) TEOAE (same, absent) DPOAE (same, absent) ABR (same, wave I was absent bilaterally, but the latencies of waves III and V were within normal limits) LiSN-S (same pattern) vHIT (same)
Multiple mtDNA deletions	8	PTA (within ±10 dB)

Key: mtDNA—mitochondrial DNA; PTA—Pure Tone Audiogram; dB—decibels; LiSN-S—Listening in Spatialised Noise - Sentences; cVEMP—Cervical Vestibular-evoked Myogenic Potentials; ABR—Auditory-evoked Brainstem Response; TEOAE—Transient-evoked Otoacoustic Emission; DPOAE—Distortion Product Otoacoustic Emission; vHIT—Video Head Impulse Test; VNG—Video-nystagmography. ‘same’ indicates consistent results with no significant change across testing environments.

## Data Availability

The raw data supporting the conclusions of this article will be made available by the authors on request.

## Supplementary Materials

The following supporting information can be downloaded at: https://www.mdpi.com/article/10.3390/audiolres14030045/s1, Table S1: Participant demographics with details of multisystem factors affecting dizziness and unsteadiness.
