# Supplementary material for: Acceptability of Audiovestibular Assessment in the Home—A Patient Survey
Source: Audiol Res. 2024 Jun 20;14(3):545–53. doi: 10.3390/audiolres14030045 (PMC11200979; doi:10.3390/audiolres14030045)
Supplement: Supplementary file 1 [file audiolres-14-00045-s001.zip › audiolres-2996862-supplementary.pdf]

GENETIC DIAGNOSIS	MUTANT LOAD (%): Age-adjusted blood m.3243A>G heteroplasmy	MUTANT LOAD (%): Blood	MUTANT LOAD (%): Urine	MUTANT LOAD (%): Muscle	Sum completed NMDAS (25)	PHENOTYPE: MIDD	PHENOTYPE: MERRF	PHENOTYPE: MELAS	PHENOTYPE: SANDO	PHENOTYPE: Kearns-Sayre syndrome	PHENOTYPE: Leigh syndrome	PHENOTYPE: Asymptomatic	PHENOTYPE: Other	Vestibular symptoms reported?	Migraine	Previous Stroke	Ataxia	Hearing	Vision	SENSATION (foot and ankle): Temperature	SENSATION (foot and ankle): Pain	SENSATION (foot and ankle): Light touch	Muscle Strength	Fatigue	FRAMEWORK DIAGNOSIS	NEURO-OTOLOGY DIAGNOSIS
	DIAGNOSIS														MULTISYSTEM FACTORS AFFECTING BALANCE AND DIZZINESS											
m.3243A>G, *MT-TL1*	38	8		78	14								Y	Y			Y	bSNHL	NL	√	√	-	NL	Y	VM	VD
m.3243A>G, *MT-TL1*	34	12	31		35								Y	Y			Y	bSNHL	NL	√	√	- (L)	-		Nil	Nil
m.3243A>G, *MT-TL1*	35	7	32		25								Y	Y	Y		Y	bSNHL	R	√	√	- (L)	-		Nil	VD
m.3243A>G, *MT-TL1*	32	9			13	Y								Y				bSNHL	NL	√	√	√	NL		VD	VD
m.3243A>G, *MT-TL1*	35	5			23	Y								Y				bSNHL	DR	√	-	-	-		VD	Nil
m.3243A>G, *MT-TL1*	11	3			15	Y								Y				NL	NL	√	√	- (L)	NL		VM	VM
m.3243A>G, *MT-TL1*	57	14			12	Y								Y		Y		bSNHL	M	√	√	√	NL		Nil	VD
m.3243A>G, *MT-TL1*	91	38			13								Y		Y			bSNHL	M	√	√	-	NL		VM	Nil
m.3243A>G, *MT-TL1*	73	11			15	Y								Y				bSNHL	NL	√	√	√	NL		VD	VD
m.3243A>G, *MT-TL1*	100	66	73		3								Y		Y			uSNHL	NL	√	-	√	NL		Nil	Nil
Multiple mtDNA deletions					9													uCI & uSNHL	CPEO	√	√	√	NL		Nil	Nil
AD POLG					6									Y	Y			NL	NL	√	√	√	NL	Y	VM	VM
AD PEO1					20								Y	Y			Y	uSNHL	CPEO	√	√	√	-		BPPV	BPPV & VM
AR RNASEH1					28								Y	Y			Y	bSNHL	CPEO	√	√	√	NL		VD	Nil
Clinicopathological diagnosis (mtDNA mutation excluded)					14								Y	Y				NL	CPEO	√	√	√	NL		Nil	Nil
Clinicopathological diagnosis					23												Y	NL	P	√	√	√	-	Y	Nil	VD

Supplementary Information Table S1: Participant demographics with details of multisystem factors affecting dizziness and unsteadiness. (Adapted with permission from Male et al.,[10])

<u>KEY:</u> NMDAS Newcastle mitochondrial disease adult scale, mtDNA mitochondrial DNA, AD autosomal dominant, AR autosomal recessive, MIDD Maternally inherited diabetes and deafness, MERRF Myoclonic epilepsy with ragged red fibres, MELAS Mitochondrial encephalomyopathy, lactic acidosis and stroke like episodes, SANDO Sensory ataxic neuropathy, dysarthria and opthalmoparesis, Y yes, √ intact, NL normal, x absent, - reduced, L left, R right, bSNHL bilateral sensorineural hearing loss, uSNHL unilateral sensorineural hearing loss, uCI unilateral cochlear implant, R retinopathy, CPEO chronic progressive external ophthalmoplegia, M maculopathy, P ptosis, DR diabetic retinopathy, VD vestibular dysfunction, VM vestibular migraine, BPPV benign paroxysmal positional vertigo, CD cerebellar dysfunction, Nil no vestibular diagnosis.
